# Effectiveness and safety of azvudine in the treatment of COVID-19 patients: a retrospective cohort study using propensity score matching

**DOI:** 10.3389/fcimb.2025.1584261

**Published:** 2025-06-18

**Authors:** Jing Zhang, Fang Wang, Ying Xie, Qianyu Li, Zhenzhen Zhu, Yuan Dong

**Affiliations:** Department of Pharmacy, Medical Security Center Stationed in the 5th Medical Center Pharmacy Room, People’s Liberation Army of China General Hospital, Beijing, China

**Keywords:** azvudine, COVID-19, propensity score matching, hospitalization duration, retrospective study

## Abstract

**Background:**

Clinical trials have demonstrated the efficacy of azvudine in alleviating clinical symptoms among patients with coronavirus disease 2019 (COVID-19). However, evidence regarding its real-world effectiveness and safety profile remains limited.

**Objective:**

To evaluate the effectiveness and safety of azvudine in COVID-19 patients.

**Methods:**

This retrospective cohort study included 192 COVID-19 patients hospitalized in Fengtai District, Beijing, from November 1 to December 31, 2022. Patients were divided into azvudine (n=118) and non-azvudine (n=74) groups. Propensity score matching (PSM) was applied to balance baseline characteristics (age, sex, vaccination status, etc.), yielding 48 matched pairs. Outcomes included time to SARS-CoV-2 RNA negativity, hospitalization duration, and symptom resolution (fever, cough). Adverse events were recorded.

**Results:**

After PSM, 48 pairs of COVID-19 patients were identified. The azvudine group exhibited significantly shorter hospitalization than the non-azvudine group (median: 8 vs. 10 days, *P ≤* 0.05). No significant differences were observed in time to RNA negativity (4.23 vs. 4.52 days, *P*>0.05), fever duration (2 vs. 2 days, *P*>0.05), or cough duration (4.5 vs. 5 days, *P*>0.05). One case of mild gastrointestinal discomfort was reported in the azvudine group.

**Conclusion:**

Azvudine significantly reduced hospitalization duration in mild-to-moderate COVID-19 patients with a favorable safety profile.

## 1 Introduction

From the start of COVID-19 until November 10, 2024, over 776.8 million confirmed COVID-19 cases and over 7 million confirmed deaths were notified to WHO across 234 countries. To date, more than 1,000 variants of SARS-CoV-2 have been identified, and the virus epidemic continues to pose a significant threat to global health (Yu and Chang, 2022). Currently, Antiviral agents against COVID-19 reported mainly include polymerase inhibitors, protease inhibitors, inhibitors of nucleoside and nucleotide reverse transcriptase, entry and uncoating inhibitors, and other antivirals (Yuan et al., 2023).

Azvudine is the world’s first dual-target anti-HIV drug (Zhang et al., 2021), which also exhibits certain activity against HCV (Smith et al., 2009), EV71 (Xu et al., 2020), and HBV (Zhou et al., 2012). It’s also the first small molecule anti-SARS-CoV-2 therapeutic drug, which was independently developed in China (Ma and Chen, 2023). It is a nucleoside analogue of the viral RNA-dependent RNA polymerase (RdRp), a well-known conserved protein without a corresponding human protein, making it an ideal target for treating SARS-CoV-2 (Maio et al., 2021; Wang et al., 2021). RdRp is metabolized intracellularly into an active 5 ′-triphosphate metabolite. This activator acts exclusively on RdRp to embed viral RNA during SARS-CoV-2 RNA synthesis. Thus, inhibition of SARS-CoV-2 replication achieves a therapeutic effect for COVID-19 (Chen and Tian, 2023). During 2020-2022, four phase III clinical trials were carried out to evaluate the efficacy and safety of azvudine in treating COVID-19. The results demonstrated that azvudine reduced the nucleic acid negative conversion time, viral load, and the time to improvement of clinical symptoms in patients with moderate COVID-19. Moreover, azvudine has a favorable safety and tolerability profile (Zhu, 2023). In 2022, It was conditionally approved in China for COVID-19 treatment; and on August 9, 2022, it was included in the 2019 Coronary Virus Disease Diagnosis and Treatment Guidelines (Ninth Edition) issued by the National Health and Health Commission and the State Administration of Traditional Chinese Medicine. In the same year, it was approved by the National Healthcare Security Administration on 12 August for inclusion in the medical reimbursement list.

Current guidelines prioritize the use of direct antiviral drugs in COVID-19 patients, and several real-world studies have demonstrated that early antiviral treatment reduces the risk of serious illness. A real-life cohort study confirmed that Remdesivir has a good safety profile and significantly reduces the risk of disease progression and COVID-19 sequelae compared to untreated controls, in the SARS-CoV-2 vaccination and Omicron era, in patients at high risk of developing severe disease (Mazzitelli et al., 2023). A Single-Center, Prospective, Comparative, Real-Life Study proved that Nirmatrelvir/Ritonavir and Remdesivir show equivalent efficacy in preventing hospitalization and death (Basoulis et al., 2023). A Chinese real-world retrospective cohort study conducted a head-to-head comparison between azvudine and Nirmatrelvir/Ritonavir. Azvudine demonstrated comparable safety and potential clinical advantages in specific outcomes (Wei et al., 2023). However, the current evidence regarding the effectiveness and safety of antiviral agents remains inadequate. In this study, we aimed to evaluate the real-world clinical effectiveness and safety of azvudine in hospitalized COVID-19 patients at the Medical Security Center Stationed in the 5th Medical Center, PLA General Hospital.

## 2 Methods

### 2.1 Study design and patient selection

A retrospective cohort study included 192 COVID-19 patients(confirmed via RT-PCR or antigen testing)hospitalized in Fengtai District, Beijing between November 1 and December 31, 2022. Inclusion criteria (Department of Medical Emergency Response and National Health Commission of the PRC, 2023): (1)age≧18 years; (2)All patients diagnosed with COVID-19 were confirmed by testing positive for SARS-CoV-2 real-time polymerase chain reaction, SARS-CoV-2 antigen, or both; (3) taking only one type of antiviral drug, azvudine, or not taking any antiviral drug; (4) hospitalization days≧3 days. Exclusion criteria: (1) receiving other antiviral agents such as Nirmatrelvir/Ritonavir, Remdesivir or Lopinavir/Ritonavir; (2) pregnancy/lactation; (3) patients concurrently enrolled in other interventional clinical studies; (4) cases presenting incomplete or missing critical baseline parameters; (5) individuals with documented hypersensitivity to azvudine or excipients contained in the pharmaceutical formulation; (6) subjects exhibiting moderate-to-severe hepatic insufficiency (Child-Pugh class B/C) or renal dysfunction.

Patients were stratified into azvudine (n=118) and non-azvudine (n=74) groups.

### 2.2 Interventions

Patients in both groups received standard symptomatic care. The control group received standard care primarily aimed at alleviating clinical symptoms. Specifically: (1) Patients with high fever underwent physical cooling measures with antipyretic medication administration; (2) Those manifesting severe cough and sputum production were administered antitussive and expectorant agents. Meanwhile, the azvudine group received antiviral treatment with azvudine tablets: swallowed whole on an empty stomach, 5mg each time, once a day, until two nucleic acid conversions 24 hours apart, for a maximum of 14 days.

### 2.3 Data collection

The following patient data were systematically collected: (1) age and gender; (2) vaccination status; (3) preexisting comorbidities; (4) clinical typing of COVID-19; (5) symptomatic treatment regimens; (6) duration of febrile symptoms and cough(days); (7) viral clearance time-defined as the interval between initial positive RT-PCR result and first consecutive negative test; (8) length of hospital stay (admission to discharge interval); (9) azvudine-related adverse events(AEs).

The primary endpoints included virological clearance time and hospitalization duration (Gao et al., 2023). Viral shedding cessation was confirmed according to the Diagnosis and Treatment Protocol for COVID-19 (Trial Version 9.0), requiring either: a) Two consecutive negative SARS-CoV-2 RT-PCR tests (cycle threshold [CT] value≥35 for both ORF1ab and N genes, with≥24-hour sampling interval); b) CT values>35 for O and N genes. Secondary endpoints focused on the resolution of clinical symptoms, particularly the duration of pyrexia and persistent cough.

### 2.4 PSM

To address potential confounding factors and mitigate baseline imbalances between comparative cohorts in real-world study settings, PSM was systematically implemented using SPSS Statistics software(version 25.0; IBM Corporation, Armonk, NY, USA). The propensity score model incorporated clinically relevant baseline covariates including chronological age, biological sex, comorbidity burden, vaccination status, COVID-19 clinical staging, administration of symptomatic treatment, presence of febrile symptoms, occurrence of cough, and cycle threshold (Ct) values of admission nucleic acid testing. Ct values upon admission constituted predictor covariates. The azvudine-treated and control cohorts underwent 1:1 nearest-neighbor matching; caliper width was set at 0.05 propensity score standard deviations.

### 2.5 Statistical analysis

Statistical analyses (Yu et al., 2023) were performed using SPSS 25.0 (IBM Corp., USA), with two-tailed *P* values ≤ 0.05 considered statistically significant. Normally distributed continuous variables were expressed as mean ± standard deviation ( $\bar{\text{x}}$ ± s) and compared using independent Student’s t-tests. Non-normally distributed continuous variables were reported as median (interquartile range) [M(P25, P75)] and compared through Mann-Whitney U tests. Categorical variables were presented as frequencies (percentages), with between-group differences assessed using χ^2^ tests or Fisher’s exact tests when appropriate.

## 3 Results

### 3.1 Baseline characteristics

**Figure 1:**
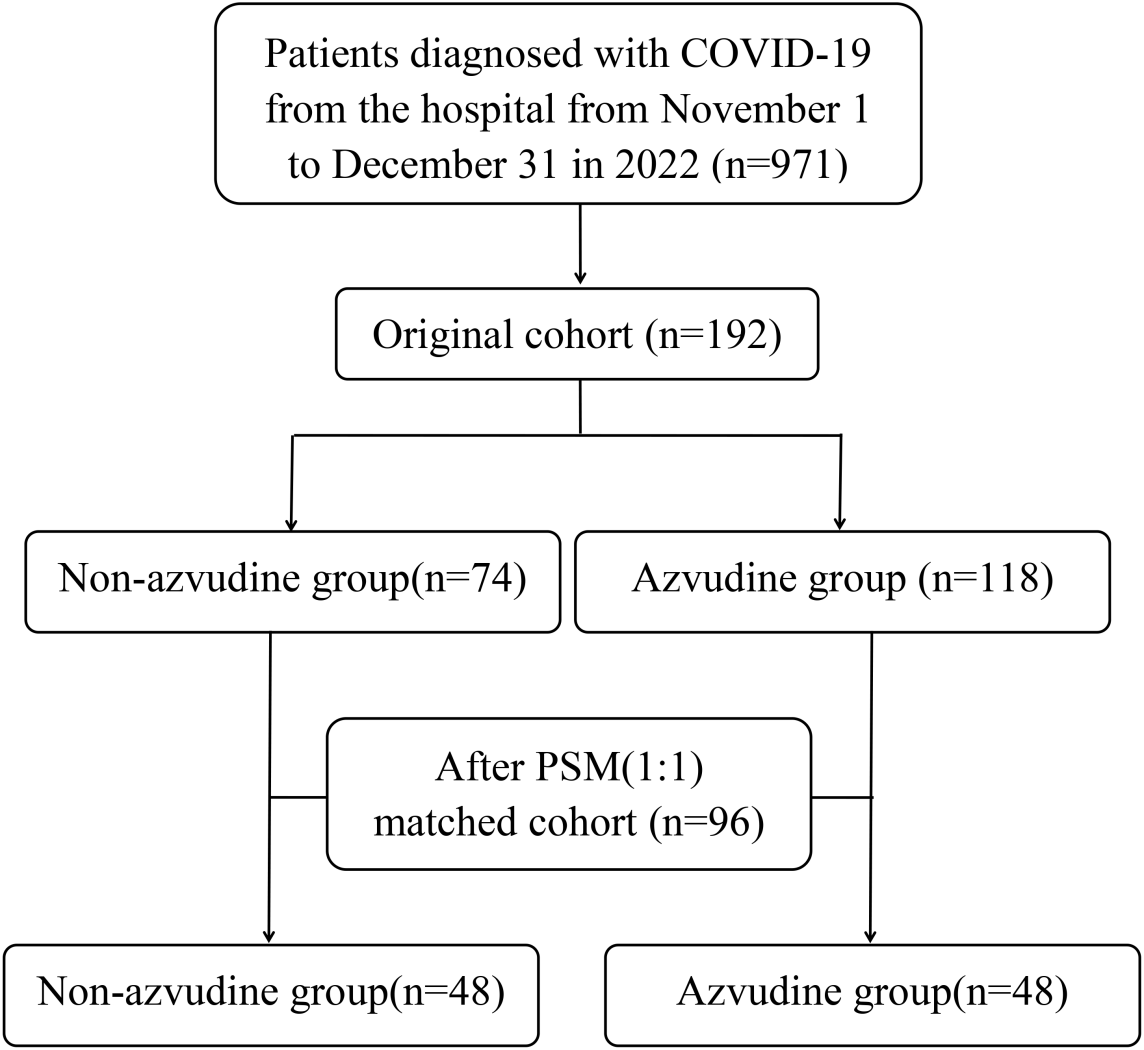
Flow chart of retrospective cohort study.

The real-world cohort initially comprised 192 eligible patients, including 118 recipients of azvudine therapy and 74 controls receiving standard symptomatic treatment only before PSM. Significant intergroup disparities were observed in baseline characteristics including age distribution, COVID-19 clinical classification, and febrile status (all *P*<0.05). Following 1:1 PSM implementation, balanced cohorts of 48 matched pairs were successfully generated (**Figure 1**). Comprehensive pre- and post-matching demographic and clinical characteristics are systematically presented in **Table 1** for both groups.

**Table 1:** Comparison of baseline data before and after matching between the two groups of patients.

Variables	Before PSM (n/%)				After PSM (n/%)				
	Non-azvudine n=74	Azvudine n=118	t/Z/χ2	*P* value	Non-azvudine n=48	Azvudine n=48	t/Z/χ2	*P* value	
Gender	Male:37 (50.0)	64 (54.2)	0.328	0.567	23 (47.9)	24 (50.0)	0.042	0.838	
	Female:37 (50.0)	54 (45.8)			25 (52.1)	24 (50.0)			
Age	53.5 (48.8, 58.5)	38.5 (28.0, 54.3)	–	0.000	53.0 (48.0, 60.8)	54.5 (45.8, 65.0)	–	0.829	
Comorbidity burden	Yes:25 (33.8)	32 (27.1)	0.968	0.325	19 (39.6)	18 (37.5)	0.044	0.834	
	No:49 (66.2)	86 (72.9)			29 (60.4)	30 (62.5)			
Vaccination status	Yes:55 (74.3)	72 (61.0)	4.175	0.124	30 (62.5)	30 (62.5)	0.232	0.890	
	No:4 (5.4)	6 (5.1)			3 (6.3)	2 (4.2)			
	Unspecified:15 (20.3)	40 (33.9)			15 (31.3)	16 (33.3)			
Clinical classifications	Mild:69 (93.2)	95 (80.5)	5.921	0.020	43 (89.6)	45 (93.8)	0.545	0.714	
	Moderate:5 (6.8)	23 (19.5)			5 (10.4)	3 (6.3)			
Administration of symptomatic treatment	Yes:73 (98.6)	114 (96.6)	0.745	0.651	47 (97.9)	48 (100.0)	1.011	1.000	
	No:1 (1.4)	4 (3.4)			1 (2.1)	0 (0.0)			
Presence of febrile symptoms	Yes:49 (66.2)	96 (81.4)	5.639	0.018	34 (70.8)	37 (77.1)	0.487	0.485	
	No:25 (33.8)	22 (18.6)			14 (29.2)	11 (22.9)			
Occurrence of cough	Yes:48 (64.9)	88 (74.6)	2.076	0.150	31 (64.6)	35 (72.9)	0.776	0.378	
	No:26 (35.1)	30 (25.4)			17 (35.4)	13 (27.1)			
Ct value at admission	31.8 (29.6, 33.5)	31.7 (29.8, 33.6)	–	0.965	31.57 ± 2.41	31.60 ± 2.60	-0.052	0.959	

*For continuous data with normal distribution, the statistical data is represented by the t value. For continuous data that does not follow a normal distribution, the statistics are represented by Z-values; for categorical variable data, statistics are expressed as χ^2^-values.

In the original cohort, participants in the non-azvudine group exhibited a significantly higher mean age than those in the azvudine group (mean age 53.5 vs. 38.5, *P*<0.001). Additionally, significant intergroup disparities were observed in COVID-19 clinical severity classifications and febrile symptoms. However, after PSM, no statistically significant differences in baseline characteristics, including subgroup distributions, were identified between the non-azvudine and azvudine cohorts.

### 3.2 Comparison of the viral nucleic acid conversion time and hospitalization duration

The primary endpoints were Viral nucleic acid conversion time and hospitalization duration. The mean time to conversion in the azvudine group was 4.23 days (vs. 4.52 days in the non-azvudine group). The median time to conversion in the azvudine group was 4 (3, 5.75) days and 4 (2, 7) days in the non-azvudine group, with no statistically significant intergroup difference (*P*>0.05) (**Table 2**). However, the hospitalization duration in the azvudine group was significantly shorter than that in the non-azvudine group(median: 8 vs. 10; *P ≤* 0.05), indicating that azvudine may reduce hospital stays for COVID-19 patients (**Table 2**).

**Table 2:** Primary endpoints [M(Q1,Q3)].

Groups	Cases	The time to viral nucleic acid conversion/d	Hospitalization duration/d
Non-azvudine group	48	4 (2, 7)	10 (7, 11)
Azvudine group	48	4 (3, 5.75)	8 (6, 10)
		*P*=0.416	*P*=0.032

### 3.3 Comparison of fever and cough duration

Secondary endpoints included the duration of fever and cough. The azvudine group exhibited a trend toward shorter cough duration compared to the non-azvudine group post-treatment, though the difference lacked statistical significance (*P* > 0.05). Similarly, no significant advantage in fever duration was observed between the groups (**Table 3**).

**Table 3:** Secondary endpoints [M(Q1,Q3)].

Groups	Cases	The days of fever/d	The days of cough/d
Non-azvudine group	48	2 (0, 3)	5 (0, 7)
Azvudine group	48	2 (1, 3)	4.5 (0, 7)
		*P*=0.881	*P*=0.794

### 3.4 Safety assessment

Among azvudine-treated patients, one case of epigastric discomfort and loose yellow stools was reported. This patient concurrently received Ibuprofen sustained-release capsules, Lanqin oral liquid, human interferon α2a for injection, and Watermelon Cream Runlaryngeal tablet. As per the azvudine tablet prescribing information, gastrointestinal adverse reactions (e.g., diarrhea, nausea, abdominal pain) have been documented in clinical trials. Although causality could not be definitively excluded, symptoms resolved following symptomatic treatment with berberine hydrochloride tablets and montmorillonite powder.

## 4 Discussion

Few real-world studies have evaluated azvudine’s effectiveness and safety. This retrospective propensity score-matched analysis of COVID-19 patients (November–December 2022) demonstrated that azvudine significantly reduced hospitalization duration compared to the non-azvudine group, despite no significant differences in viral conversion time, fever duration, and cough duration. These findings align with a prior retrospective study by Zhang et al. (2023) which reported shorter hospital stays in 170 azvudine-treated patients. Notably, no severe adverse reactions were observed in our cohort; only one mild gastrointestinal event occurred, resolving post-treatment. A 2022 Phase III trial in China further supports azvudine’s safety profile. Among 341 participants, azvudine and placebo groups exhibited comparable rates and severity of adverse events (AEs), with most graded as mild to moderate (CTCAE v4.03). Grade 3 AEs occurred in one azvudine recipient versus three placebo recipients, and no Grade 4 or serious AEs were reported in the azvudine group.

This study further validates that mild and moderate COVID-19 patients can benefit clinically from taking azvudine. Anyway, this study has several limitations: (1) exclusion of severe/critical cases, precluding effectiveness assessment in high-risk subgroups (9); (2) lack of laboratory or lung CT data to evaluate additional prognostic variables; (3) the study is inherently limited by its retrospective design, which precluded analysis of vaccination status impacts on treatment efficacy; (4) a relatively small sample size. Larger prospective studies are warranted to validate these findings. Despite these constraints, this analysis provides valuable insights into azvudine’s role in COVID-19 management.

## 5 Conclusion

The results of our study indicated that azvudine significantly shortened the patients hospitalization duration. There were no significant differences in nucleic acid conversion time, fever duration, and cough duration.

## Data Availability

The original contributions presented in the study are included in the article/supplementary material. Further inquiries can be directed to the corresponding author/s.
